# The *B*. *subtilis* Accessory Helicase PcrA Facilitates DNA Replication through Transcription Units

**DOI:** 10.1371/journal.pgen.1005289

**Published:** 2015-06-12

**Authors:** Christopher N. Merrikh, Bonita J. Brewer, Houra Merrikh

**Affiliations:** 1 Department of Microbiology, University of Washington, Seattle, Washington, United States of America; 2 Department of Genome Sciences, University of Washington, Seattle, Washington, United States of America; University of Geneva Medical School, SWITZERLAND

## Abstract

In bacteria the concurrence of DNA replication and transcription leads to potentially deleterious encounters between the two machineries, which can occur in either the head-on (lagging strand genes) or co-directional (leading strand genes) orientations. These conflicts lead to replication fork stalling and can destabilize the genome. Both eukaryotic and prokaryotic cells possess resolution factors that reduce the severity of these encounters. Though *Escherichia coli* accessory helicases have been implicated in the mitigation of head-on conflicts, direct evidence of these proteins mitigating co-directional conflicts is lacking. Furthermore, the endogenous chromosomal regions where these helicases act, and the mechanism of recruitment, have not been identified. We show that the essential *Bacillus subtilis* accessory helicase PcrA aids replication progression through protein coding genes of both head-on and co-directional orientations, as well as rRNA and tRNA genes. ChIP-Seq experiments show that co-directional conflicts at highly transcribed rRNA, tRNA, and head-on protein coding genes are major targets of PcrA activity on the chromosome. Partial depletion of PcrA renders cells extremely sensitive to head-on conflicts, linking the essential function of PcrA to conflict resolution. Furthermore, ablating PcrA’s ATPase/helicase activity simultaneously increases its association with conflict regions, while incapacitating its ability to mitigate conflicts, and leads to cell death. In contrast, disruption of PcrA’s C-terminal RNA polymerase interaction domain does not impact its ability to mitigate conflicts between replication and transcription, its association with conflict regions, or cell survival. Altogether, this work establishes PcrA as an essential factor involved in mitigating transcription-replication conflicts and identifies chromosomal regions where it routinely acts. As both conflicts and accessory helicases are found in all domains of life, these results are broadly relevant.

## Author Summary

In bacteria the concurrence of DNA replication and transcription leads to potentially deleterious encounters between the two machineries. These encounters can destabilize the genome and lead to mutations. Both eukaryotic and prokaryotic cells possess conflict resolution factors that reduce the detrimental effects of these collisions. In this study we show that without the essential *Bacillus subtilis* accessory DNA helicase, PcrA, the replication machinery slows down at certain regions of the chromosome in a transcription-dependent manner. PcrA is essential to life but incomplete depletion of PcrA only partially inhibits cell survival. We find that, under these conditions, partial survival defects are significantly exacerbated in the presence of a single severe conflict. In summary our work identifies a high degree of conservation for accessory helicase function in conflict resolution, directly establishes PcrA’s role in co-directional conflict resolution, and maps the natural chromosomal regions where such activities are routinely needed. Because both conflicts and accessory helicases are found in all domains of life, the results of this work are broadly relevant.

## Introduction

Transcription is a major impediment to DNA replication. Head-on conflicts arise when a gene is encoded on the lagging strand, prompting transcription in the direction opposite to the movement of the replisome. Conversely, transcription of genes encoded on the leading strand causes co-directional conflicts which occur between replication and transcription complexes moving in the same direction. The significantly faster rate of replication, relative to transcription, leads to the meeting of the two complexes co-directionally. Though the deleterious effects of head-on replication-transcription conflicts have been appreciated for some time, the impact of the less severe, but more common, co-directional conflicts has only recently been established [1–6].

The majority of genes in bacterial genomes are co-oriented with replication [7–11]. This genome co-orientation bias is thought to be a strategy to avoid the more deleterious head-on replication-transcription conflicts. Although the co-orientation bias of bacterial genomes reduces the prevalence of head-on conflicts, by definition, it increases the prevalence of co-directional genes. Importantly, most highly transcribed and essential genes, including rRNA and tRNA operons, are co-oriented with replication [7–9,11–15]. Previously identified consequences of co-directional conflicts at rRNA genes include replication stalling and restart in *Bacillus subtilis* [5]. In *Escherichia coli*, co-directional conflicts caused by permanently arrested RNA polymerases have been shown to cause double-strand breaks [6].

Cells possess mechanisms that promote replication progression through conflict regions [16]. One strategy is the use of accessory helicases such as *E*. *coli* UvrD, Rep, and DinG [17]. Previous work has shed light on the beneficial effects of accessory helicases on replisome progression. There is strong evidence showing that in *E*. *coli* these proteins can promote replisome progression through artificially inverted (head-on) rRNA genes and that their combined activities contribute to cell survival under these conditions [17]. Additional work showed that mutations in RNA polymerase that impact the expression of rRNA genes (rpoB* mutations) can rescue the viability of strains lacking Rep and UvrD, implying that accessory helicases may also function at endogenous co-directionally oriented rRNA genes [18]. However, direct evidence that accessory helicases resolve co-directional conflicts is lacking.

The bulk of previous reports have focused on the rRNA genes, except for one instance where inversion of a large region of the chromosome containing several protein-coding genes of both head-on and co-directional orientations also led to growth defects in a *rep uvrD* double mutant [17]. Though these data implied that protein-coding genes may also produce physiologically relevant replication-transcription conflicts, they did not dissect the contribution, if any, that co-directional conflicts played in the associated growth defect. Because co-directional genes represent at least 50% of most bacterial genomes, and are known to cause replisome stalling in *B*. *subtilis*, the additive impact of many co-directional conflicts, especially at rRNA or highly expressed protein-coding genes, may be quite significant. Therefore, clarifying the relative impact of accessory helicases in otherwise identical head-on versus co-directional conflicts should yield insight into their impact on replication. Moreover, identifying the regions where accessory helicases act within the genome should clarify their predominant function(s) in the cell.

Homologues of the *E*. *coli* accessory helicases UvrD and Rep exist in all domains of life. *B*. *subtilis*, which diverged from *E*. *coli* more than 1 billion years ago [19], harbors one such homologue, PcrA [20,21]. Whereas Δ*uvrD* and Δ*rep* are only lethal in *E*. *coli* in combination, deletion of *pcrA* alone is lethal in *B*. *subtilis*. Currently, the reason PcrA is essential remains unclear, however the lethality of both Δ*uvrD* Δ*rep* and Δ*pcrA* strains can be rescued by inactivation of the RecFOR pathway. This complex facilitates the loading of RecA onto single stranded DNA gaps [22,23]. PcrA and UvrD can remove RecA from DNA [24] and PcrA depletion strains are hyper-recombinogenic [21]. These findings suggest that the essential nature of these helicases is related to excessive RecA activity. It is unclear whether the RecA removal activity of UvrD is important in the context of conflicts. Additionally, it is unclear whether the conflict resolution activity of UvrD is conserved in PcrA.

Several studies have shown that both *E*. *coli* UvrD and *B*. *subtilis* PcrA interact with RNA polymerase [25,26]. However, the physiological significance of these interactions remains unclear, except for a role recently identified for UvrD in transcription-coupled repair [27]. Furthermore, although there is an abundance of *in vitro* studies on UvrD and PcrA’s helicase and ATPase activities, which are coupled, the physiological relevance of these functions *in vivo* are poorly understood [24,28,29]. The physiological significance of PcrA’s RNA polymerase interaction or helicase/ATPase activities is unclear. Additionally, whether these features of accessory helicases are important in conflict resolution is unknown.

Here we show that PcrA associates with both head-on and co-directional genes and reduces transcription-dependent replisome stalling at these regions. Using chromatin immunoprecipitations (ChIPs) of the replicative helicase DnaC and 2D gel analyses, we were able to detect increased replisome stalling at a single conflict in both the head-on and co-directional orientations when PcrA is depleted. Accordingly, partial depletion of PcrA, which is normally sub-lethal, causes a severe survival defect when a single head-on gene is highly transcribed. Using ChIP-Seq of DnaC and PcrA we identified chromosomal regions where PcrA predominantly associates and impacts replisome stalling. These regions include the heavily transcribed rRNA, tRNA, and other co-directionally and head-on oriented protein-coding genes. Additionally, we found that the helicase/ATPase activity of PcrA, but not its C-terminal RNA polymerase interaction domain, is required for survival in general. Furthermore, although its recruitment is not ablated, a helicase/ATPase mutant of PcrA cannot mitigate conflicts and shows dominant negative effects on replisome stalling at specific transcription units. Altogether, these results identify PcrA as an essential conflict mitigation factor, provide direct evidence for its activity in replication progression through co-directional genes, map the endogenous regions of the chromosome where PcrA routinely resolves conflicts, and establish a correlation between PcrA’s essential function and its helicase/ATPase activity in resolution of conflicts.

## Results

### Construction and characterization of a conditional PcrA degradation mutant

**Fig 1:**
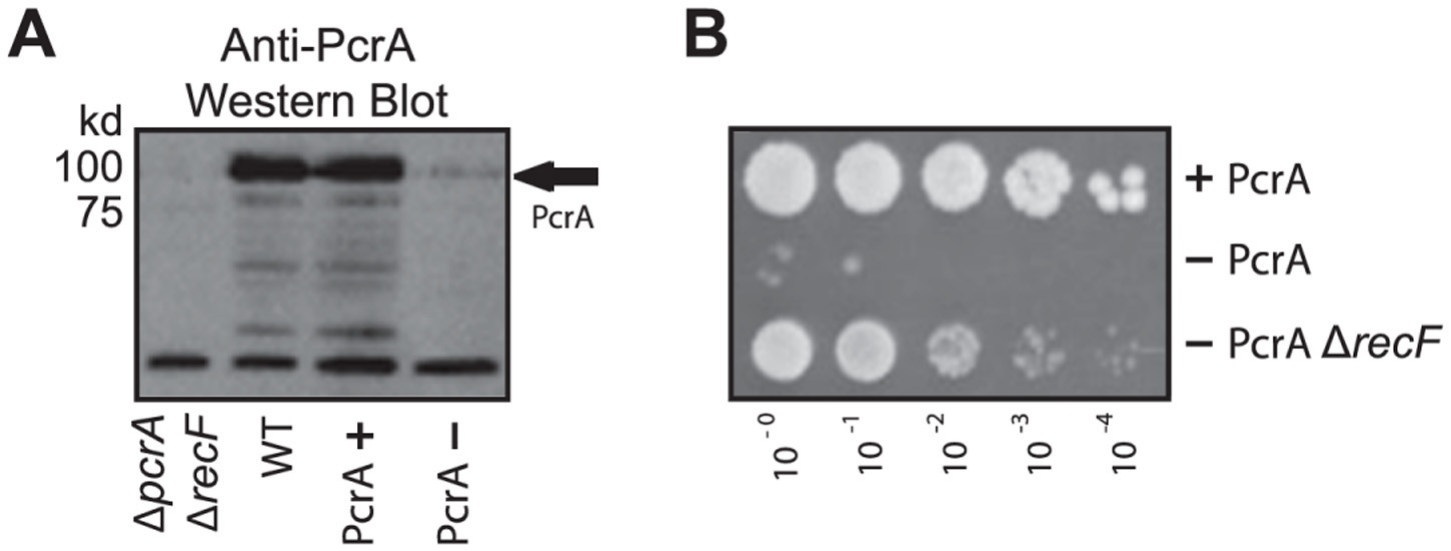
A conditional depletion system for PcrA. A) Fusion protein PcrA-ssrA is conditionally depleted following transcriptional induction of the adaptor protein gene *sspB*, via 100 μM IPTG treatment. A representative western blot probed for native PcrA shows approximately 90% depletion following IPTG addition (PcrA- vs. PcrA+) versus the non-specific band near the bottom of the gel which was used as a loading control. The black arrow indicates the location of PcrA on the blot. PcrA levels in a wild-type strain are shown in lane 2 (WT), and are equivalent to levels in the degron strain prior to depletion (PcrA+). Specificity of the polyclonal anti-PcrA antibody is demonstrated by the lack of signal in a *pcrA* deletion strain, which is suppressed by *recF* deletion. B) PcrA depletion (on plates containing 100 μM IPTG) is lethal, and is rescued by *recF* deletion. Top row: Negative control strain harboring P*spank*-*sspB* only. Middle row: PcrA Degron strain harboring both P*spank*-*sspB* and *pcrA-ssrA*. Lower row: PcrA degron and Δ*recF*.

To investigate whether PcrA might mitigate replication-transcription conflicts in *B*. *subtilis*, we generated a conditional mutant by developing a PcrA degron strain as previously described [30]. In this strain, the C-terminal end of the endogenous *pcrA* gene is translationally fused to an *ssrA* degradation tag. At a second locus, we integrated an IPTG-inducible gene encoding the SspB adaptor protein. PcrA is depleted when IPTG is added to the culture, inducing the expression of SspB, which then binds the ssrA tag and delivers PcrA to the ClpXP protease. After treatment of cells with 100 μM IPTG for 15 minutes we observe a 60–90% depletion of PcrA (Fig 1A). Under these conditions cell survival is completely ablated (Fig 1B), indicating that our conditional depletion system functions as expected. To further validate that our PcrA depletion mimics a complete *pcrA* knockout, we also tested the ability of *recF* deletion to rescue viability defects of PcrA depleted cells. We found that PcrA depletion in the absence of *recF* no longer causes viability defects, consistent with previous studies (Fig 1B, and [21]).

### Depletion of PcrA leads to increased replisome stalling at transcription units of both orientations

If PcrA mitigates conflicts, then replisome progression should be hindered in the absence of PcrA. We previously showed that ChIP of the replicative helicase DnaC is sensitive enough to identify replisome stalling and restart at both head-on and co-directional genes [5]. Because the majority of the genes within the genome are co-directional, and we are most interested in the physiologically relevant and naturally occurring conflicts, we again chose to use this technique for our studies.

**Fig 2:**
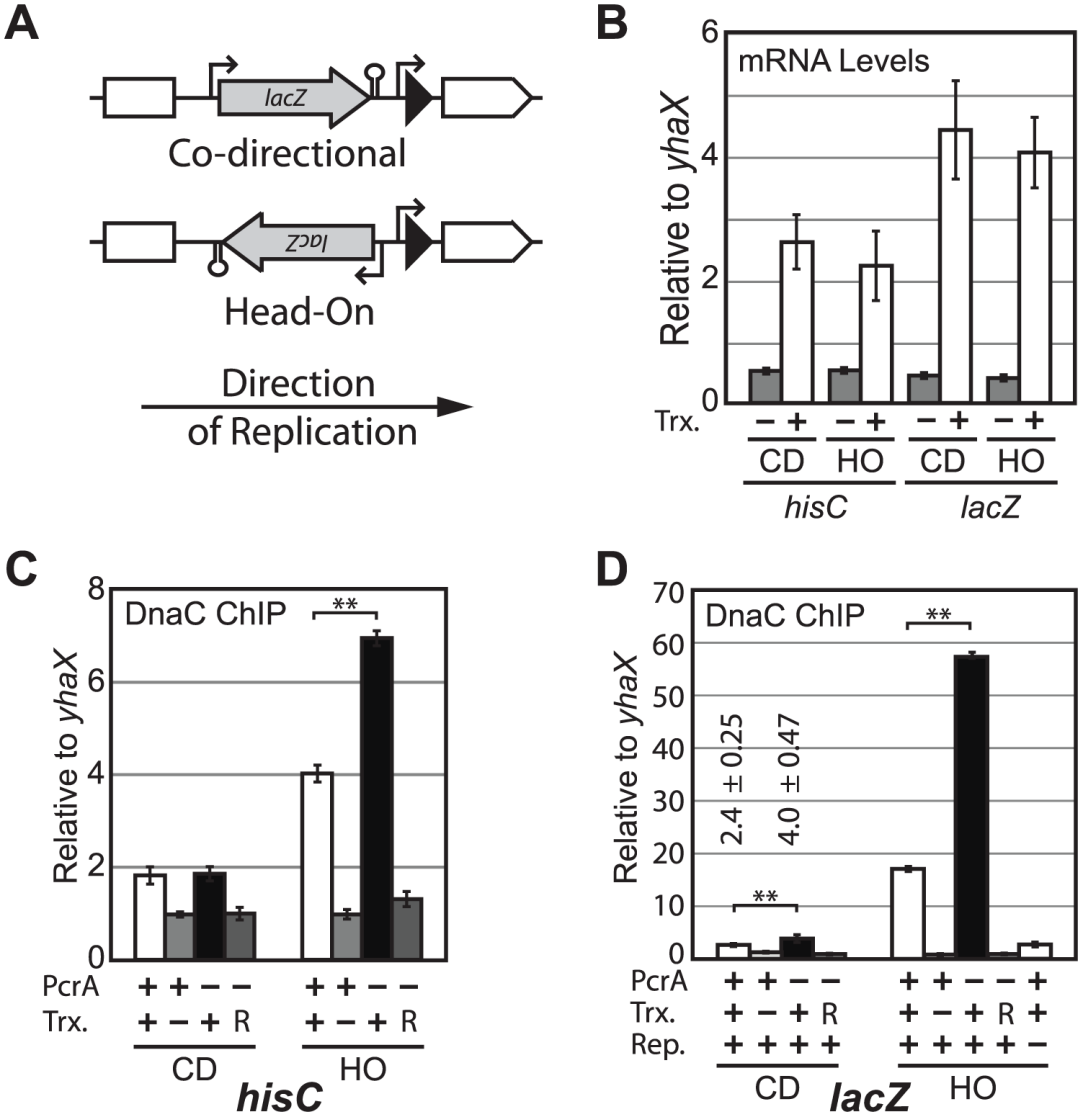
PcrA reduces DnaC association with engineered conflict regions. A) The P*xis*-*lacZ* reporter (gray arrow) and MLS resistance gene (black triangle) were integrated onto the chromosome either co-directionally (CD), or head-on (HO) to replication at the *thrC* locus (upstream and downstream *thrC* fragments used for integration into the chromosome are shown in white). P*spank(hy)*-*hisC* constructs share the same conceptual design, but have a spectinomycin resistance gene in place of the MLS gene and are integrated at *amyE*. B) mRNA levels were determined by RT-qPCR using primers that bind in the middle of *hisC* or *lacZ*. Levels are shown relative to the control gene, *yhaX*. “Trx” refers to transcription before or after induction with IPTG (Trx- and Trx+, respectively). C) The relative association of DnaC with either CD or HO *hisC* was determined by ChIP-qPCR and plotted relative to its association with control region *yhaX*. D) The relative association of DnaC with *lacZ* was determined as in 2C. Here *lacZ* is expressed/repressed in strains lacking/possessing repressor protein ImmR, respectively. “R” refers to inhibition of transcription by subsequent addition of rifampicin). “Rep.” refers to unperturbed or HPUra-inhibited replication. An additional condition is shown where DnaC association with *lacZ* was determined after replication was inhibited by 15 minutes of HPUra treatment (last bar on the right in the *lacZ* “HO” panel).

To test the potential role of PcrA in conflict mitigation, we used DnaC ChIPs to measure replisome stalling at two inducible genes: *hisC* under the lower strength, IPTG-inducible P*spank*(*hy*) promoter and *lacZ* under the strongly expressed P*xis* promoter, which is constitutively active or repressed, depending upon the strain (Fig 2A and 2B). These constructs were integrated into the chromosome in either the head-on (HO) or co-directional (CD) orientation relative to replication. To account for the possibility that local context might affect our experiments, we used two different integration loci: P*xis*-*lacZ* was integrated at *thrC* (left chromosomal arm) and P*spank*(*hy*)-*hisC* was integrated at *amyE* (right chromosomal arm). These constructs each allow for the direct comparison of otherwise identical co-directional and head-on conflicts. The two loci, and distinct coding genes, also control for potential chromosomal location-dependent and gene sequence-dependent effects.

Using ChIP-qPCR we measured the degree of DnaC association with the regions expressing either *lacZ* or *hisC* in the two orientations, when transcription is activated or repressed, and in the presence or absence of PcrA. We observed a transcription-dependent enrichment of DnaC with both the head-on and co-directional *lacZ* and *hisC* genes relative to a previously established control region, *yhaX* ([5,31–33] and several other control loci around the chromosome give similar results to *yhaX* [34]) (Fig 2C and 2D). Normalization of ChIP data from a region of interest compared to a control locus (in this case *yhaX*) generally provides the most consistent results between experiments. However, we also analyzed non-normalized, raw IP/input values to rule out any potential artifacts of normalization in these experiments. Induction of transcription leads to a significant increase in DnaC association with the head-on *lacZ* construct regardless of normalization to *yhaX*, though the absolute degree of enrichment varies between experiments (S1A Fig). Consistent with the higher transcriptional level of *lacZ*, DnaC association at head-on *lacZ* was approximately 4-fold higher than at head-on *hisC* (Fig 2B, 2C and 2D). To measure the impact of PcrA on replisome stalling we carried out DnaC ChIPs in strains harboring the PcrA degron system. After depletion of PcrA, DnaC association increased significantly relative to the conditions where PcrA was present at wild-type levels, with both of the head-on reporters (Fig 2C and 2D, (and see S1B Fig) for non-normalized IP/Input values in the degron experiment). Again, the effect of PcrA depletion on replisome stalling was less severe at *hisC* relative to the *lacZ* gene, where DnaC association tripled, reaching 55-fold over the control region. PcrA depletion did not affect DnaC association with co-directional *hisC*, however it caused a small increase in DnaC association—from 2.4 to 4.0 (p < 0.01) with co-directional *lacZ*. To determine if the increased stalling in the absence of PcrA is due to transcription, after we induced the conflict by de-repressing transcription, we treated cells with 300 μg/ml rifampicin for 3 minutes to inhibit transcription initiation. We observed that DnaC association both before and after PcrA depletion decreased to baseline after rifampicin treatment, indicating that the increased replisome stalling at these conflict regions, without PcrA, is due to replication-transcription conflicts (Fig 2C and 2D).

Previous reports have demonstrated that certain proteins may artificially stick to transcription units and produce ChIP artifacts [35,36]. To control for the possibility that DnaC could bind non-specifically during ChIPs, we shut off replication for 15 minutes through the addition of HPUra. HPUra is a specific nucleotide analogue that inhibits PolC by inserting into its active site [37]. Under these conditions DnaC association with *lacZ* drops by approximately 90%, demonstrating that this signal is replication-dependent (Fig 2D). In contrast, a 15 minute HPUra treatment does not reduce the association of RNA polymerase beta subunit (RpoB) with this reporter region, as determined with RpoB ChIPs (S2 Fig). Therefore DnaC ChIP signal is unlikely to be an artifact of transcription.

**Fig 3:**
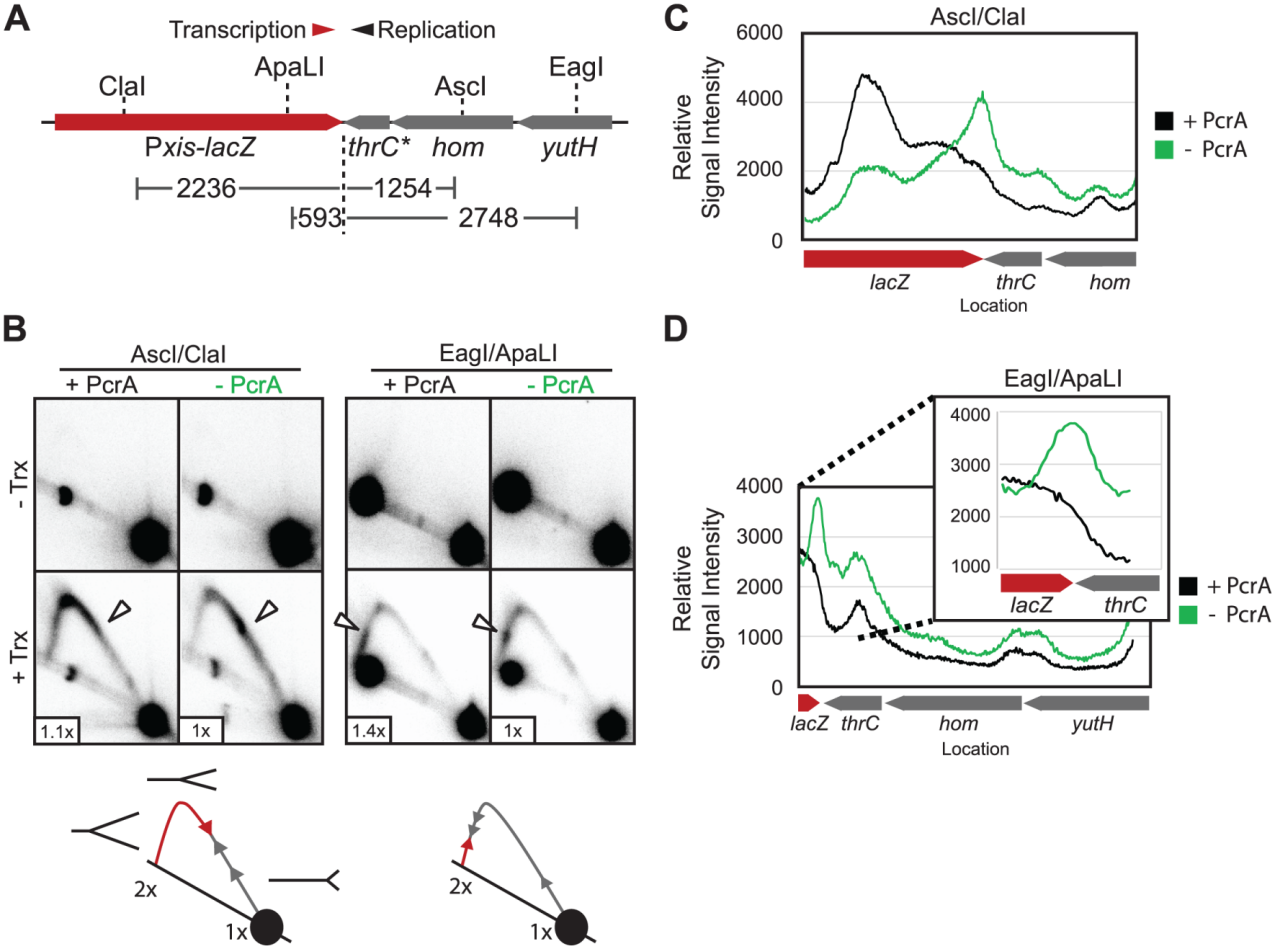
Replication intermediates accumulate at conflict sites. A) Genomic DNA from PcrA degron strains HM876 (Trx-), or HM877 (Trx+) was enzymatically digested for 2D gel analysis: two discrete fragments were produced, both of which encoded the 3’ end of the *lacZ* gene which was targeted for qPCR analysis in Fig 2. The location (in base pairs) of the *lacZ* 3’ end within each fragment is indicated. B) 2D gel analysis of the AscI/ClaI or EagI/ApaLI digested fragment containing the *lacZ* 3`end and surrounding region. The approximate location of the *lacZ* 3`end is indicated with a white arrow, and the relative 1N spot intensity is indicated at the lower left (useful as a loading control). An incomplete digestion product (black spot) is present along the arc of linears in the EagI/ApaLI digest. C) Quantification of signal intensity along the Y-arc of the AscI/ClaI digestion fragment. D) Quantificaiton of the Y-arc of the EagI/ApaLI fragment. A magnified view of the region of the arc corresponding to the 3`end of *lacZ* is shown at the right.

To further confirm the results of our ChIP assay we analyzed replication fork stalling at head-on oriented P*xis*-*lacZ* using 2D gels (Fig 3). 2D gels allow for the direct visualization and analysis of replication intermediates [38]. Restriction digestion (Fig 3A) of replicating chromosomes releases branched fragments that generate Y-arcs on 2D gels (Fig 3B diagram). The 2D gels with *lacZ* fail to reveal any replication intermediates in the absence of transcription (Fig 3B, top panels)—with or without PcrA—because replication through the transcriptionally silent region of the chromosome is extremely fast. However, after transcriptional induction an arc of replication intermediates forms, consistent with impaired replication fork movement approaching and entering the *lacZ* gene. The comparison between the +PcrA and—PcrA gels reveals that the signal intensity is not uniform across the Y arcs, with areas of pausing indicated by locally darkened regions (Fig 3B lower panels). Quantification of the Y-arc demonstrates that the increase in replication fork stalling at the 3`end of *lacZ* is roughly 1.7 ± 0.25 following PcrA depletion (Fig 3C and 3D). These results indicate that replication is indeed slowed by the head-on transcription of *lacZ*, and that depletion of PcrA exacerbates this effect. We also quantified the apparent decrease in signal within the region of the larger Y-intermediates 5`of the initial stall site: Signal in this region drops as low as ~ 0.64 ± 0.13 when PcrA is depleted (PcrA-/PcrA+). We note that a digestion intermediate is present on the EagI/ApaLI gels, partly obscuring the *lacZ* region. These undigested DNAs produced a second Y-arc that is not obscured and showed the same trends displayed in 3B on the right hand side, and 3D (S3 Fig). These data suggest that, absent PcrA, replication forks are highly compromised in their ability to proceed past the initial point of contact with head-on RNA polymerases. They also serve as confirmation of our ChIP experiments which indicated that that DnaC accumulates at the 3`end of head-on genes after PcrA depletion (Fig 2).

### PcrA associates with conflict regions

**Fig 4:**
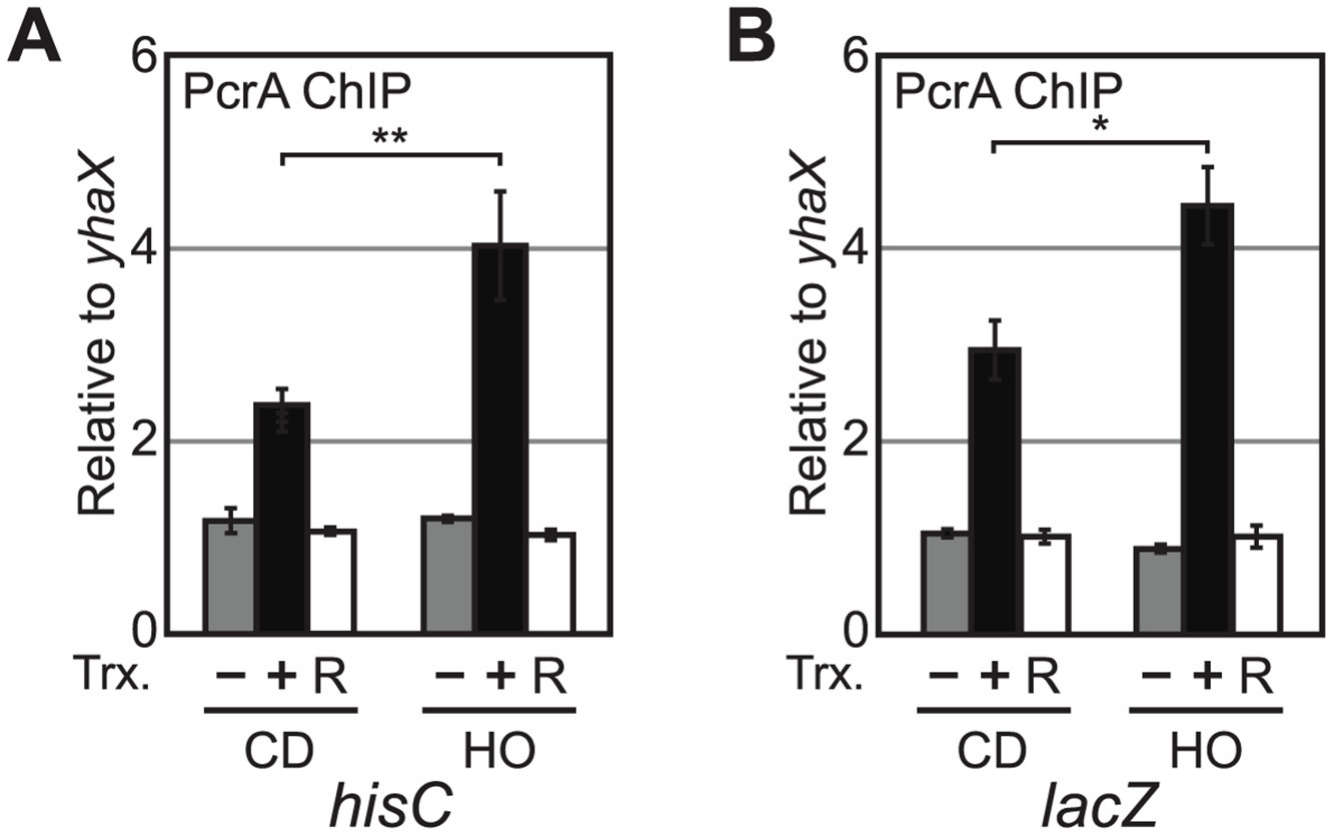
PcrA associates with engineered conflict regions. A) PcrA association was determined by ChIP of PcrA followed by qPCR for *hisC* in the presence (+IPTG) or absence (-IPTG) of transcription (Trx+, or Trx-, respectively) and following transcriptional induction and subsequent inhibition by rifampicin (Trx- “R”). B) ChIP-PCR of PcrA was performed as in 4A, but for *lacZ*. *P<0.05 and **P<0.01.

It is conceivable that the effects of PcrA depletion on replisome stalling is indirect. If PcrA acts directly at our reporters, it should be physically present there. To address this possibility, we tested the association of an N-terminally Myc-tagged PcrA with our reporters by ChIP-qPCR. After confirming that Myc-tagged PcrA compliments the cell death phenotype observed in the degron (S4 Fig), we performed ChIP in our reporter strains using a monoclonal antibody specific for the Myc peptide. We observed that PcrA associates with both *hisC* and *lacZ* reporters after transcription induction (Fig 4A and 4B). Similar to DnaC, PcrA also associates more with both head-on reporters compared to their co-directional counterparts.

To further confirm that PcrA association with the conflict region depends on transcription, we treated cells with rifampicin for 3 minutes. Because rifampicin inhibits transcription initiation, the RNA polymerase occupancy within the gene significantly decreases after treatment, thereby preventing the occurrence of replication-transcription conflicts. We found that addition of rifampicin reduced PcrA association with the *hisC* and *lacZ* loci to background levels (Fig 4A). These findings suggest that PcrA association with a given conflict region depends on transcription.

### Depletion of PcrA leads to increased replisome stalling at endogenous chromosomal loci

**Fig 5:**
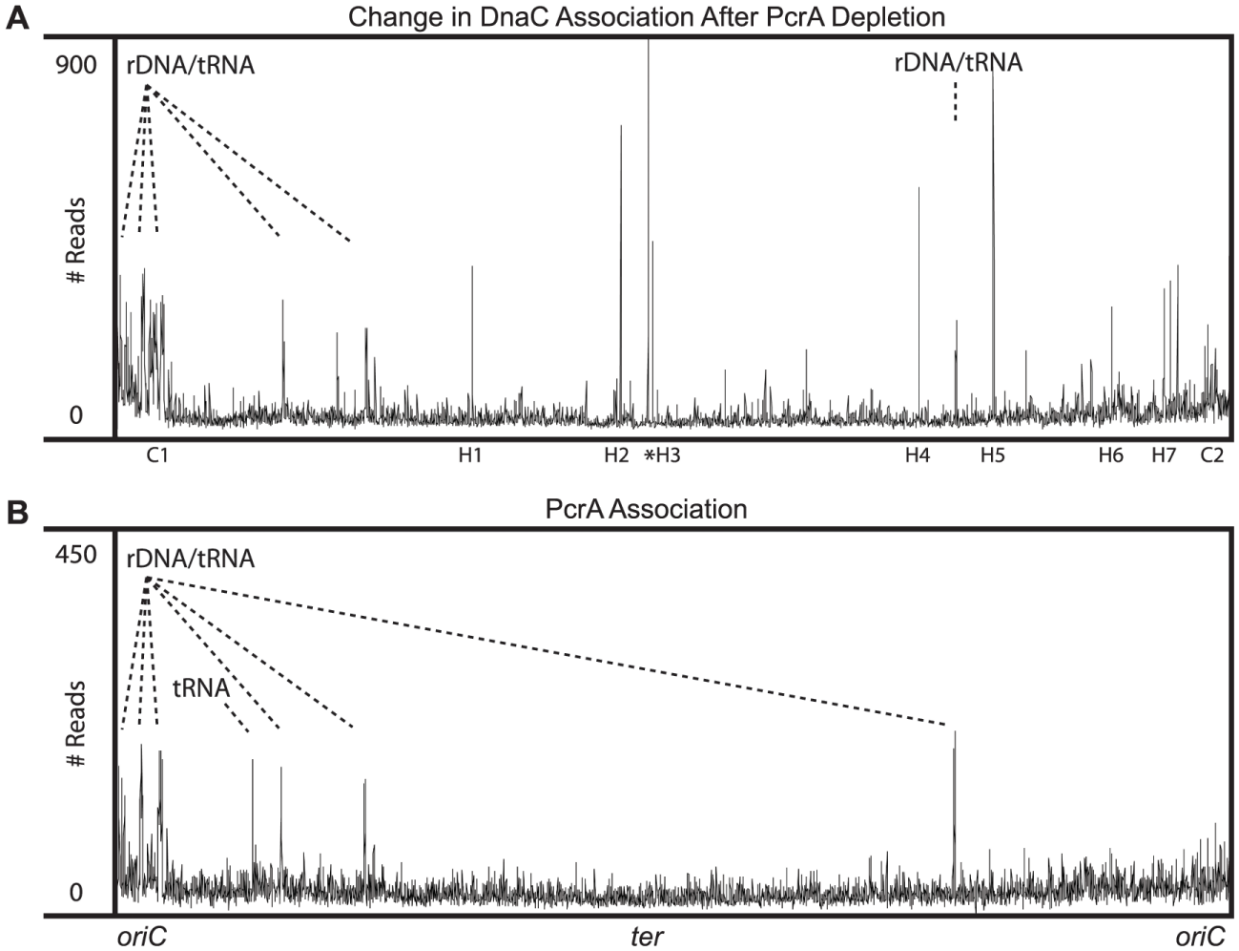
PcrA associates with, and reduces DnaC association with, endogenous conflict regions. A) ChIP-Seq data showing chromosomal locations where DnaC association increases after PcrA depletion (strain HM448). Data were calculated by first normalizing ChIP-Seq samples to inputs. Normalized ChIP signal when PcrA was present was then subtracted from ChIP signal after PcrA depletion. The resulting differential signal is shown. Peaks at rRNA genes and tRNA genes are indicated. Selected peaks are labelled according to orientation. C = co-directional, H = head-on: ribosomal protein genes at 8–13° position (C1), *ssbA* (C2), *pit* (H1), *cotC* (H2), *yoaM* (H3), *rpsD* (H4), *thrC* (H5), *amtB* (H6), *dltA-E* (H7). The *ter* region is indicated by *. B) ChIP-Seq of Myc-PcrA in an otherwise wild-type strain containing the *myc-pcrA* allele (HM224). ChIP data were normalized to input, then non-specific peaks (identified via antibody control ChIP-Seq) were subtracted out. Peaks at rRNA and tRNA genes are indicated.

We set out to identify endogenous chromosomal regions where PcrA promotes replication progression. To globally examine all chromosomal regions, we conducted DnaC ChIP-Seq experiments in the PcrA degron strain (HM448). After deep sequencing both the total (input) DNA used for the IPs, and the DNA recovered from the DnaC ChIPs, we normalized the ChIP sequence reads to the input signal for both the +PcrA (-IPTG) and the—PcrA (+IPTG) samples by subtracting input from IP signal. We then subtracted the normalized-IPTG signal from the normalized +IPTG signal, identifying the regions where DnaC association increases after PcrA depletion. We found that following PcrA depletion, DnaC association increased significantly at several regions including the rRNA and tRNA genes, and many protein-coding genes of both orientations (Fig 5A and S5 Fig). For comparison, non-normalized DnaC ChIP-Seq data are displayed in S6 Fig. In addition to the rRNA and tRNA genes, the most prominent peaks included the head-on operons *dltABCDE*, *amtB/glnk*, *ykaA-pit*, the head-on ribosomal protein gene *rpsD*, and the co-directional ribosomal protein genes encoded between *rrnW* and *rrnI* (from 8°-13°) (Fig 5A and S5 Fig). As expected, activation of the degron system also led to a significant increase in DnaC association downstream of the *thrC* locus where P*spank-sspB* produces an artificial head-on conflict (Fig 5A, peak H5).

Consistent with the observation that PcrA promotes replication across transcription units, we frequently observed an increase in ChIP signal throughout whole genes. Therefore, rather than calling peaks, we found it most appropriate to simply identify genes affected by PcrA depletion. To do this, we calculated the ChIP signal in terms of the maximum signal, or area under the curve within each gene on the chromosome. To account for peaks present in intergenic regions, we performed the same analysis with all intergenic regions greater than 5 nucleotides in length. Genes containing ChIP signal with a maximum height of more than 5-fold over background were considered peak containing regions. A list containing the 50 regions that met these criteria is presented in S1 Table. However, a comprehensive list of all genes and intergenic regions is also included in S2 Table. Interestingly, though replisome stalling was most prominent within genes, we also observed peaks within promoter regions, including the promoters for *nagP* and *qdoI* which contain transcriptional repressor binding sites. These data are consistent with reports that PcrA removes DNA binding proteins in addition to RNA polymerase [28] Among the gene regions affected by PcrA activity, approximately 16% (8 genes) are head-on, and 84% (42 genes) were co-directional. These data demonstrate that replisome progression slows within genes of both head-on and co-directional orientation, consistent with DnaC association measurements at the *lacZ* reporter genes.

Previous reports have suggested that ChIP may produce inaccurate data due to non-specific association of target proteins with the rRNA genes. To assess the accuracy of our DnaC ChIP signal at endogenous rRNA genes, we analyzed the formation of replication intermediates at these regions using 2D gels (S8 Fig). When PcrA is present we did not observe any replication intermediates within rRNA genes, despite our use of digest conditions that allowed us to collectively probe for all 10 rDNA repeats simultaneously (S8 Fig, left side). However, after PcrA depletion we clearly observed the formation of replication intermediates within these regions (S8 Fig, right side). This result is consistent with our DnaC ChIP-Seq data and demonstrates that when PcrA is absent, replication slows when it passes through the co-directionally oriented rRNA genes. This strongly suggests that the DnaC ChIP-Seq data is accurate and not an artifact caused by the non-specific adhesion of DnaC proteins to rDNA or other genes.

We also considered the possibility that the activity of PcrA at conflict regions could be related to the removal of RecFOR-loaded RecA—a function of PcrA that has been well-characterized *in vitro*. To test this possibility we carried out the DnaC ChIPs-qPCRs, with and without PcrA, in a Δ*recF* background and measured the levels of DnaC association at the most common conflict region we identified—rRNA genes. We found that even when RecF is not present, depletion of PcrA leads to increased DnaC association with rRNA loci (S9 Fig). This result suggests that any effects of RecF related to PcrA activity in conflicts occur either downstream of replisome stalling or are independent of conflicts.

### PcrA associates with rRNA and tRNA genes

In keeping with the results of our reporter assays, we anticipated that on the chromosome PcrA should associate with the regions where replisome stalling increases following PcrA depletion. To identify these regions we conducted ChIP-Seq of Myc-PcrA in strain HM224 (Fig 5B). This strain differs from the PcrA degron strain (HM448) used in Fig 5A in that it does not possess the *sspB* gene encoded at *thrC*. To reduce background signal due to non-specific interaction of the Myc antibody with endogenous proteins, we normalized Myc-PcrA ChIP signal to both input DNA and a mock IP with the anti-Myc antibody. We first normalized both the PcrA ChIP and mock IP to their respective input (total) DNA data sets by subtracting the input signal from the IP signal at each nucleotide position. Furthermore, because this normalization still produced nonspecific signal (peaks present in both the experimental and mock IPs) we also subtracted the mock IP-total signal from the PcrA ChIP-total signal. For comparison, non-normalized and normalized data are shown together in S7 Fig. The resulting data set indicated that PcrA associates predominantly with the rRNA and tRNA genes (Fig 5A). We quantified the data as with the DnaC ChIP-Seq data set, and present a list of peak-containing regions in S3 Table. A comprehensive list of all gene regions can be found in S4 Table. The absence of detectible signal at protein-coding genes identified in the DnaC ChIP-Seq following PcrA depletion suggests that either the association of PcrA with these loci is simply below our detection limit or that its activity at these regions is transient.

### PcrA association and activity at endogenous conflict regions is transcription-dependent

**Fig 6:**
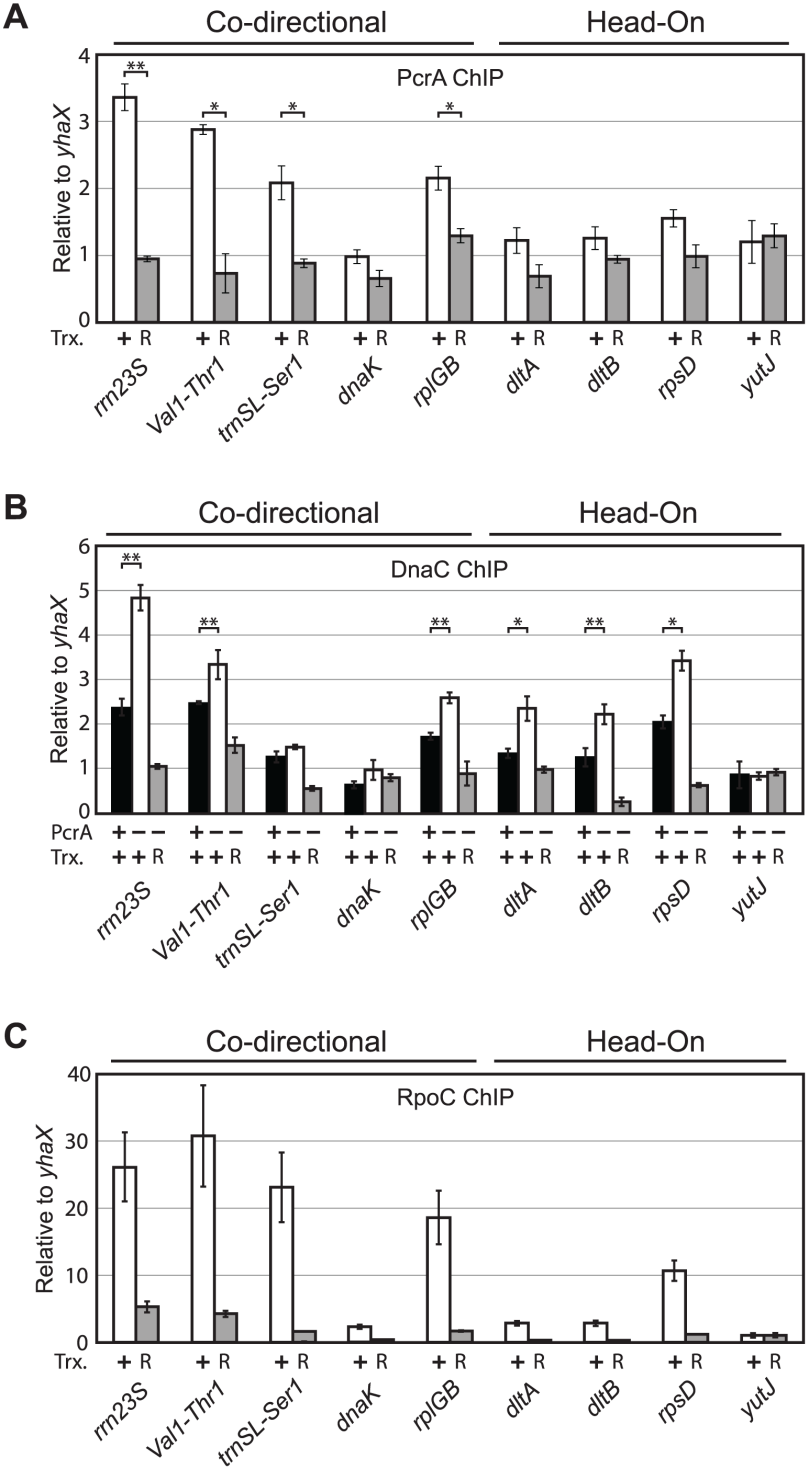
PcrA and DnaC association with specific chromosomal regions are transcription-dependent and correlate with RpoC association. A) Relative association of PcrA with nine candidate loci, compared to the control locus *yhaX* was measured by ChIP-qPCR, with active (white bars, before rifampicin treatment) and inhibited (gray bars, after 3 min. of rifampicin treatment) transcription. B) Relative association of DnaC (compared to *yhaX*) as measured by ChIP-qPCR in the presence of PcrA and transcription (no rifampicin, black), following PcrA depletion (no rifampicin, white), and following PcrA depletion and transcription shut off (3 min. of rifampicin treatment, gray bars). C) Relative association of RpoC-GFP with the candidate regions (compared to *yhaX*) was measured by ChIP-qPCR before (white) and after transcription inhibition with rifampicin (gray). Co-directional and head-on genes are indicated above the graph. Pearson correlation coefficient for DnaC (-PcrA) vs. PcrA association = 0.7709. Pearson correlation coefficient for co-directional genes: R = 0.7 for DnaC (-PcrA) vs. RpoC, and R = 0.9 for PcrA vs. RpoC. N ≥ 5. *P<0.05 and **P<0.01.

We set out to determine if PcrA association and activity at the endogenous loci we identified is transcription-dependent. To address this question and to confirm our ChIP-Seq data we analyzed DnaC and PcrA association with different candidate loci relative to the control locus *yhaX* using ChIP-qPCRs (Fig 6). The candidate loci included rRNA, tRNA and protein-coding genes of both orientations. Specifically, for genes in the co-directional orientation, we examined the ribosomal RNA gene *rrn23S*, the *Val1*-*Thr1* tRNA pair, *trnSL*-*Ser1*, and the protein coding gene *rplGB*. Though we did not detect PcrA association with *rplGB* in our PcrA ChIP-Seq experiment, DnaC levels increased at this locus following PcrA depletion. Therefore, *rplGB* was anticipated to represent the lower end of our detection range for PcrA association. For genes in the head-on orientation, we examined the ribosomal protein gene *rpsD* and two genes from the *dltA-E* operon, *dltA* and *dltB*. (We also confirmed DnaC association, with and without PcrA, with the genes *pit*, *cotC*, and *yoaM*—see S10 Fig). As a negative control, we also investigated the head-on gene *yutJ* which showed no detectable PcrA or DnaC association.

To determine if the association of DnaC and PcrA with the candidate loci was transcription-dependent we used rifampicin to shut off transcription initiation: we treated cells for 3 minutes then analyzed PcrA and DnaC association with these regions. Consistent with the ChIP-Seq experiments, we found that PcrA association with some coding genes was low in ChIP-qPCR experiments (Fig 6A). However, regardless of the degree of association, transcription shut-off reduced association of PcrA with all examined loci to some degree, except at the negative control locus, *yutJ* (Fig 6A). Also in agreement with our ChIP-Seq data, in the ChIP-qPCR experiments, DnaC association increased after PcrA depletion at all chromosomal loci examined, with the exceptions of the single tRNA gene *trn-SL-ser1*, *dnaK* and *yutJ* (Fig 6B,S10 Fig and see S11 Fig for non-normalized IP/Input values for DnaC association with the rRNA genes). Presumably, a single tRNA gene may simply be so short that local RNA polymerase occupancy remains limited, thereby minimizing the impact on replication. Regardless, we do observe stalling at a locus encoding multiple tRNA genes (*Val1-Thr1*). As with the PcrA ChIPs, after rifampicin treatment and in the absence of PcrA, DnaC association with the other loci was ablated (Fig 6B). These data confirm that PcrA’s association and activity at conflict regions requires active transcription as initially indicated by our reporter data.

### RNA polymerase occupancy correlates with PcrA and DnaC association

To determine if the association and activity of PcrA was correlated with transcription, we took a second approach: we measured RNA polymerase occupancy at the loci identified in the ChIP-Seq experiments (as a measure of transcription level) by conducting ChIP-qPCRs of a GFP-fusion allele of the beta’ subunit of RNA polymerase, RpoC, using an anti-GFP polyclonal antibody. We found that RpoC associates at predictably varying degrees with all but the negative control candidate regions tested (Fig 6C). As anticipated, rifampicin treatment reduced this association at all examined loci by 80% or more (Fig 6C). To control for potential artifacts of the GFP-fusion we carried out ChIP-qPCRs of the beta subunit of RNA polymerase, RpoB, using a native antibody. Although the absolute degree of association of RpoB compared to the RpoC ChIPs was different, the relative association patterns with conflict regions were equivalent (S12 Fig).

RpoC ChIP-qPCRs allowed us to compare RNA polymerase occupancy with PcrA association and conflict severity (DnaC association). RpoC, DnaC and PcrA associations closely correlated with all co-directional genes examined (Pearson coefficient 0.7 for DnaC (-PcrA) vs. RpoC, and 0.9 for PcrA vs. RpoC). Among the head-on genes replisome stalling in the absence of PcrA correlates with PcrA occupancy (Pearson coefficient 0.89 for DnaC ChIP—PcrA vs. +PcrA ChIP) and is highest at the gene with the highest RNA polymerase occupancy, *rpsD*. This correlation suggests that conflict severity is related to transcription levels for head-on genes. We also find comparison of the co-directional and head-on genes to be informative: replisome stalling is similar between *dltA*, *dltB* and the co-directional *rplGB* gene despite the significantly lower transcription levels for *dltA* and *dltB* (roughly 3 fold lower RpoC association compared to *rplGB*). Similarly, replisome stalling at *rpsD* (head-on) is equivalent to levels at the *Val1-Thr1* tRNA genes (co-directional) despite an approximately 3 fold lower RpoC association with *rpsD*. Therefore, these data are consistent with our reporter experiments where we observed that a head-on conflict causes significantly more replisome stalling than an equivalent co-directional conflict (Fig 2C and 2D). Furthermore, the results of our genome-wide analyses, together with the results of the engineered conflict experiments, underscore the potent effects of head-on relative to co-directional conflicts. Although the signals observed in these experiments are relatively small, altogether, the consistency between the data from the various experiments shows that PcrA resolves both head-on and co-directional conflicts genome-wide.

### Partial depletion of PcrA renders cells highly sensitive to head-on conflicts

**Fig 7:**
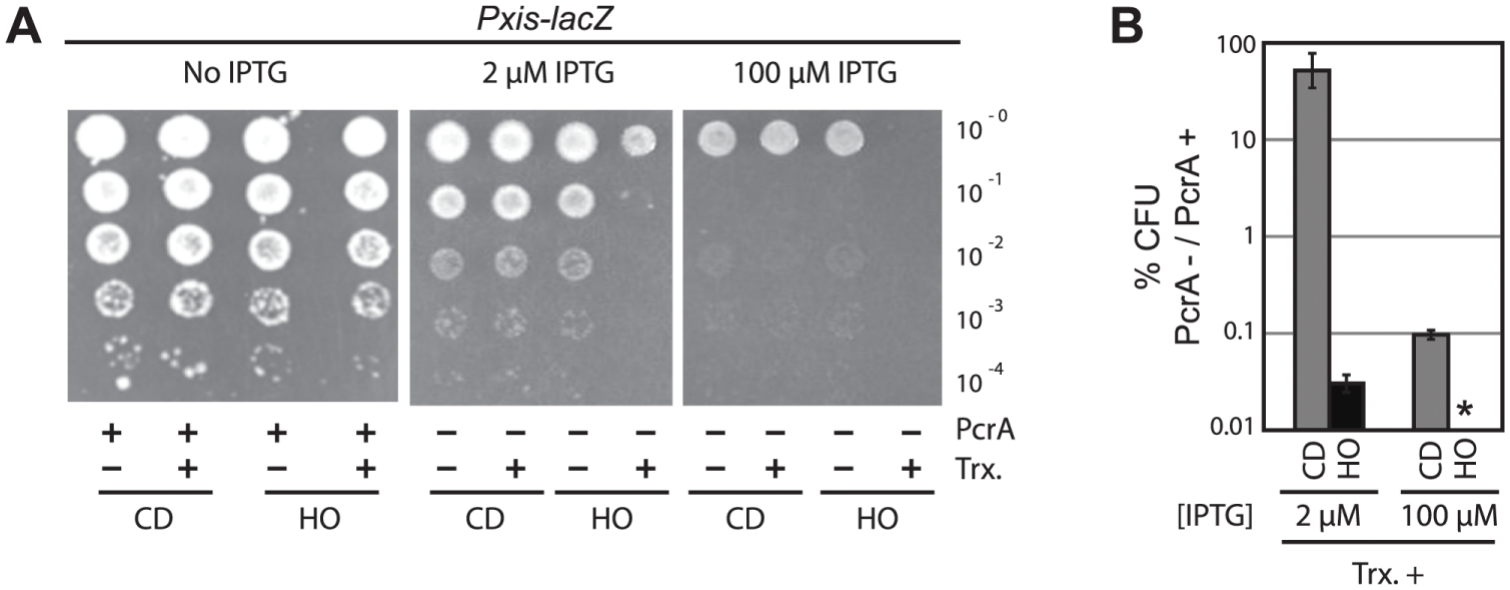
PcrA is required for survival in the presence of severe head-on conflicts. A) Plating efficiency assays were carried out for strains containing the P*xis*-*lacZ* reporter constructs. 1:10 dilutions of exponential cultures were plated on agar plates with IPTG (at 2 μM or 100 μM, as indicated, leading to PcrA depletion) or without IPTG (no PcrA depletion). Transcription repression (Trx-, strain HM876) or de-repression (Trx+, strain HM877) due to the presence or absence, respectively, of the ImmR repressor protein is indicated below. Co-directional (CD) and head-on (HO) orientations of the reporters are indicated below the dilution series. B) Quantification of cell survival following PcrA depletion during *lacZ* expression (Trx+) is plotted. Percent survival of each strain containing the reporters, after IPTG-induced depletion of PcrA, relative to pre-depletion, was quantified and plotted (CD P*xis*-*lacZ* (gray) and HO P*xis*-*lacZ* (black)). Symbol * indicates that no colonies were detected after PcrA depletion with 100 μM IPTG in the presence of head-on *lacZ*. N = 6.

Highly expressed co-directional genes are common in the genome. Therefore, expression of an additional co-directional gene should not have a major effect on cell viability. However, since the number of highly expressed head-on genes is limited during fast growth, we wondered if the addition of a highly expressed head-on gene would increase the sensitivity of cells to PcrA depletion. To test this hypothesis, we measured viability before and after partial PcrA depletion in cells harboring the *lacZ* reporters in both orientations. In the presence of PcrA, transcription of *lacZ* had no effect on cell survival regardless of its orientation (Fig 7A, no IPTG). However, following partial PcrA depletion with 2 μM IPTG we observed slow growth in cells with the co-directional *lacZ* gene and cells harboring the repressed head-on *lacZ* reporter (Fig 7A, 2 μM IPTG, and 7B). Expression of co-directional *lacZ* did not cause a decrease in plating efficiency, suggesting that a single additional co-directional conflict does not have a major effect on replication or growth rate. However, expression of head-on *lacZ* caused a severe decrease in plating efficiency after an otherwise non-lethal degree of PcrA depletion (Fig 7B). Strains harboring the *hisC* reporters (which are incorporated at different regions on the chromosome and are expressed under a different promoter) showed a similar asymmetric effect on plating efficiency (S13 Fig). These results suggest that mitigation of severe conflicts by PcrA is essential for viability.

### The C-terminal domain of PcrA is not required for its conflict mitigation activity

**Fig 8:**
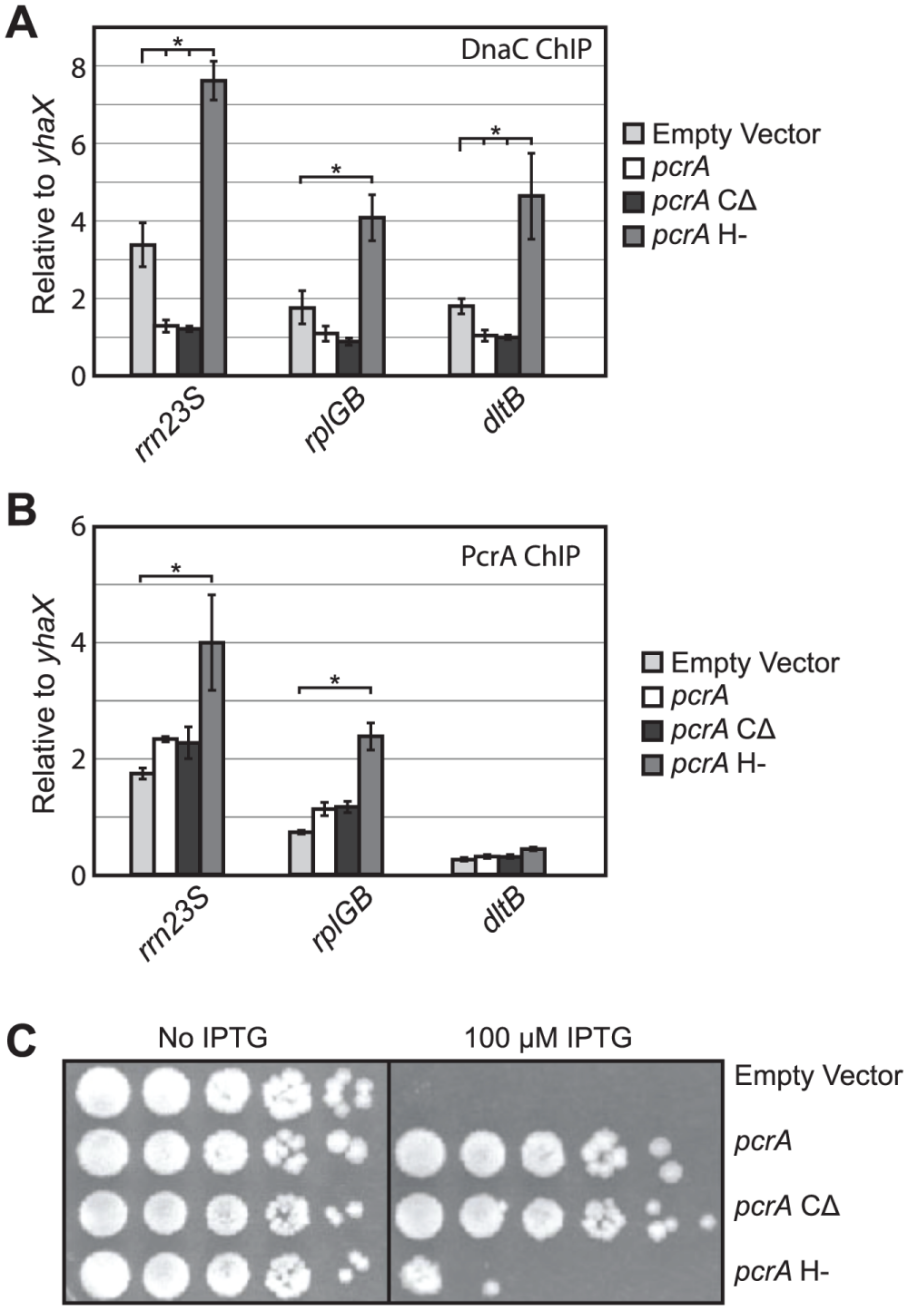
PcrA’s ATPase and helicase activity, but not its C-terminal domain, are required for conflict mitigation. A) Relative association of DnaC (compared to the control locus *yhaX*) was measured by ChIP-qPCRs. The endogenous co-directional (*rrn23S*, *rplGB*) and head-on (*dltB*) loci were analyzed without PcrA (empty vector control), in the presence of wild-type PcrA, a mutant of PcrA that should be incapable of interacting with RNA polymerase (PcrA-CΔ) or a PcrA allele lacking helicase and ATPase function (PcrA H-). B) Relative association of wild-type PcrA (compared to *yhaX*) or PcrA mutants (as well as empty vector control), as measured by ChIP-qPCRs, was determined for the same loci as in A. C) Plating efficiency assays were carried out for the PcrA degron strain expressing either an empty vector, wild-type PcrA (complementation of the PcrA degron strain), PcrA-CΔ, or PcrA H-. 10-fold dilutions of exponential cultures were plated either with (PcrA depletion at 100 μM, as indicated) or without (no PcrA depletion) IPTG. *P<0.05 and N>3.

The mechanism(s) allowing accessory helicases to be recruited to conflict regions have not been defined. PcrA could be recruited to either the replication fork or to RNA polymerase during a conflict. Previous work has established that PcrA interacts with RNA polymerase [25,26]. Whether this interaction is important for its role in conflict mitigation is unknown. To address this question, we produced a mutant allele of *myc*-*pcrA* shown to dramatically reduce PcrA’s RNA polymerase association *in vitro*, in the closely related species, *Geobacillus stearothermophilus*: *myc*-*pcrA*-ΔC [25]. This mutant lacks the final 71 amino acids of PcrA’s C-terminal domain. To avoid the potential problem of lethality, or accumulation of suppressor mutations, we expressed this mutant conditionally by placing it under the control of an IPTG-inducible promoter. When expressed in cells already harboring the PcrA degron system, the addition of IPTG triggers the simultaneous depletion of PcrA-ssrA and induction of *myc*-*pcrA*-ΔC. We found that the PcrA-ΔC protein had wild-type level activity in preventing replisome stalling and association with conflict regions of both orientations (Fig 8A and 8B). Furthermore, this mutant completely rescued the viability of PcrA degron strains, indicating that RNA polymerase interaction through the C-terminal domain of PcrA is not essential for its conflict mitigation activity (Fig 8C). It is possible that this mutant does not completely ablate PcrA’s interaction with RNA polymerase, as an N-terminal interaction between PcrA and RNA polymerase has also been detected [39]. However, based on previous work, we expect this disruption to at least partially reduce PcrA’s association with RNA polymerase. The complete lack of a phenotype in conflict mitigation and survival in strains harboring this mutant suggests that its RNA polymerase interaction is not required for PcrA’s association or activity at conflict regions.

### PcrA’s ATPase and helicase activities are required for conflict mitigation

*In vitro* studies of PcrA have demonstrated that its helicase and ATPase activities are required for its ability to separate DNA strands, but are dispensable for RecA removal. To determine if PcrA’s helicase/ATPase activities are required for its conflict mitigation functions and viability, we constructed a previously characterized separation of function allele, *myc-pcrA* K37A Q254A (PcrA H-) which is defective in helicase/ATPase activity [24]. By expressing this allele in a strain already harboring the conditional degron system (as discussed above) we were able to use ChIP-qPCR to determine whether this mutant is capable of mitigating head-on and co-directional conflicts, and measure its association with conflict sites (Fig 8). We found that the PcrA H- allele failed to resolve both head-on (*dltB*) and co-directional (*rrn23S* and *rplGB*) replication-transcription conflicts and that conflict severity at these regions was exaggerated in the presence of this mutant (Fig 8A). The inability of PcrA H- to resolve conflicts does not reflect an inability to associate with conflict sites, as we actually observed increased association of this mutant with *rrn23S* and *rplGB* (Fig 8B). This increase could potentially reflect a reduced ability to release from the DNA due to a defect in ATP hydrolysis [40]. Though we did not observe a significant association of either wild-type or the PcrA H- mutant protein at *dltB*, this result is not entirely surprising given the low PcrA ChIP signal we previously observed at these loci and the further decreased overall ChIP signal in these experiments. Nevertheless, the effect of PcrA H- on replisome stalling at *dltB* indicates that it is active at this location.

To determine if the helicase/ATPase activities of PcrA are important for viability, we carried out plating efficiency assays. Here we observed that cells depleted of the wild-type PcrA and expressing the PcrA H- allele are inviable (Fig 8C). Because this mutant is capable of removing RecA and preventing RecA-dependent strand exchange in the closely related species *S*. *aureus* [24], the loss of viability in the strain harboring the PcrA H- protein may not be due to inability to remove RecA.

## Discussion

Though the role of accessory helicases in head-on conflict resolution has been appreciated in *E*. *coli*, whether these functions are conserved in other bacteria was unknown. Furthermore, though indirect evidence has also suggested the involvement of accessory helicases in co-directional conflict mitigation, direct evidence for this role has been lacking. This gap in our knowledge is largely due to the lack of detectable replication stalling in co-directionally oriented genes using traditional methods. The use of ChIPs allowed us to overcome this difficulty and directly investigate PcrA’s activity at endogenous regions, including those that are co-directionally oriented to replication. Here we also confirm that PcrA mitigates head-on conflicts, as was anticipated based upon previous work on the *E*. *coli* homologues of PcrA. Importantly, we identify the locations where accessory helicases routinely function on the chromosome, including highly transcribed rRNA and tRNA genes, protein coding co-directional genes, and head-on genes where severe conflicts occur. The results of our studies on the separation of function mutants provide insight into the mechanism of PcrA recruitment to, and activity at, these conflict regions. Together with the plating efficiency assays, our data also strongly suggest that PcrA is essential due to its role in conflict mitigation.

### Model for PcrA recruitment to conflict regions

There are at least two models that could potentially explain how PcrA associates with the site of a conflict. Based on data from *E*. *coli* regarding the interaction and movement of Rep with the replication fork, it is conceivable that PcrA is also recruited to conflict regions via interactions with the replication fork [41]. On the other hand, reports also indicate that PcrA interacts with RNA polymerase, suggesting that PcrA may be recruited to the conflict site through this association. In our system, removing the C-terminal domain of PcrA, which prevents detectable association with RNA polymerase *in vitro*, did not impact its function in conflicts [25]. Because the data suggest that PcrA is recruited to conflict regions independent of its interaction with RNA polymerase, recruitment via an interaction with the replisome seems more plausible.

### Model for PcrA activity at conflict regions

There are at least two models for PcrA activity at conflict regions. PcrA may directly remove either RNA polymerases or RecA bound to single stranded DNA ahead of the replication fork. These two possibilities are not mutually exclusive. We found that the helicase/ATPase activities of PcrA, which facilitate strand separation, are required for conflict mitigation. This result was not necessarily expected given that mutants lacking these activities retain the ability to efficiently remove RecA from DNA in related species [24]. (As the potential RecA removal activity of PcrA H- has not yet been demonstrated in *B*. *subtilis* or *in vivo*, the following analysis is predicated on the assumption that this *in vitro* activity of *S*. *aureus* PcrA is relevant to living *B*. *subtilis* cells.)If PcrA’s role in replisome progression through transcription units stems from direct removal of RecA, then a mutant defective in helicase/ATPase activity should have, at least partially, retained the ability to mitigate conflicts. However, PcrA H- is defective in both conflict mitigation and survival. Therefore, we propose that PcrA may not mitigate conflict severity via the direct removal of RecA. In addition, the inability of this mutant to support life suggests that the rescue of *ΔpcrA* strains by inactivation of the RecFOR pathway stems from an activity of PcrA that is upstream of RecA recruitment (i.e. PcrA indirectly prevents excessive RecA recruitment). Given the correlation between decreased viability and increased conflict severity, we suspect that the essentiality of PcrA is due specifically to its activity in conflict mitigation. We prefer a model in which PcrA clears RNA polymerases ahead of the replisome, and thereby prevents excess single stranded DNA formation and subsequent RecA binding at conflict regions.

### Accessory helicases and conflict mitigation

Previous studies comparing the two types of conflicts have shown that head-on conflicts are far worse than co-directional conflicts. Furthermore, a number of studies have suggested that accessory helicases reduce the deleterious impact of transcription on replisome progression *in vivo* and *in vitro*. *In vitro* work had previously suggested that *B*. *subtilis* PcrA, similar to UvrD and Rep, promotes replisome progression past a single protein block [41]. Furthermore, PcrA can complement the survival deficiencies of UvrD and Rep mutants [20,41]. Although these studies suggested that PcrA acts similarly to UvrD and Rep, the role of PcrA in conflict resolution was not directly shown prior to this study. Furthermore, the endogenous chromosomal regions where UvrD, Rep or PcrA act have not been reported. Here, we provide direct evidence for PcrA activity in conflict mitigation and identify for the first time the natural targets of PcrA. These include, as we and others suggested, naturally occurring co-directionally oriented rDNA. Interestingly, we also identified specific head-on and co-directional genes that were not necessarily predictable.

Previous studies investigating the role of accessory helicases in conflict mitigation took advantage of severe conflicts caused by artificially inverted (head-on) rDNA, which causes major survival defects. However, the interpretations of these studies regarding conflicts may be complicated by the unique properties of rRNA genes such as GC richness, RNA polymerase stabilizing anti-termination proteins, and secondary DNA structures. Our experiments using *lacZ*, *hisC*, and several endogenous coding genes, circumvent these potential complications. Also, by detecting effects on replisome stalling at an otherwise identical gene (i.e. *hisC* or *lacZ*) in the two orientations, we can estimate the relative impact of gene expression and orientation on replisome progression: when PcrA is present, transcription-dependent replication stalling increases roughly by 6-fold at the head-on *lacZ* construct compared to its co-directional counterpart. However, in the absence of PcrA, this differential reaches more than 20-fold. Unfortunately, because these results are gathered from ensemble assays, it is difficult to further quantify the severity or frequency of replisome stalling in single cells or during a round of replication. Future experiments examining the impact of conflicts on replisome progression in single cell studies could potentially answer these questions.

### The impact of co-directional conflicts on replication

Bacterial genomes are generally organized such that highly transcribed and essential genes are oriented co-directionally with respect to DNA replication. In the case of rRNA genes, co-orientation is essentially universal. Though co-orientation reduces conflict severity, replication-transcription conflicts still occur in these regions to some degree. Our observations highlight the importance of co-directional conflicts *in vivo*, despite the apparent lack of effect on replisome progression *in vitro* [42]. Though any one co-directional conflict may not severely inhibit replication, the abundance of co-directional conflicts suggests that collectively, they can significantly slow replication. This consideration is especially important given that co-orientation appears to be a major strategy cells use to reduce conflict severity; in doing so, cells increase the impact of co-directional conflicts.

### Conflict mitigation mechanisms in different bacterial species

Although similar conflict mitigation mechanisms seem to exist in both *B*. *subtilis* and *E*. *coli*, there is a significant difference in how the two species tolerate head-on oriented rRNA genes [3,43,44]. Furthermore, the genome co-orientation bias in the two organisms is significantly different: there are many more head-on genes in *E*. *coli* compared to *B*. *subtilis* (45% vs. 26%, respectively). Together, the rDNA inversion experiments and the genome co-orientation biases from these two different species suggest that *E*. *coli* cells are much more tolerant of conflicts than *B*. *subtilis*. In keeping with these differences, studies in *E*. *coli* were unable to detect replication intermediates in rRNA genes, even after accessory helicase deletion, or rDNA inversion (replication intermediates only formed in inverted rDNAs after accessory helicase deletion) [17]. However, we observed replication intermediates in naturally occurring, co-directionally oriented rRNA genes following PcrA deletion in *B*. *subtilis*. What is the reason for the higher conflict tolerance of *E*. *coli*? Since multiple *E*. *coli* accessory helicases can be deleted without detectibly slowing replication through the rDNA, it seems likely that either *E*. *coli* possesses additional, as yet unidentified, conflict mitigation mechanisms, or that the replication machinery is inherently less susceptible to stalling at transcription units in *E*. *coli*.

## Materials and Methods

### Media and growth conditions

For all experiments, *B*. *subtilis* 168 cells were plated on LB supplemented with the corresponding antibiotics. Single colonies from plates were used to inoculate cultures of liquid rich medium (Luria-Bertain (LB)). Liquid cultures were grown to mid-log at 30°C, shaking at 260 r.p.m., then diluted back to OD 0.05, and grown again to OD 0.3–0.35 before harvesting. For Rifampicin treatments, 30 mg/ml Rifampicin in DMSO was added to a final concentration of 0.3 mg/ml, for 3 minutes.

PcrA Degron cultures were grown as above, with the exception that cells were split at OD 0.2 into two cultures, with or without IPTG at 100 μM final concentration. At OD 0.3–0.35, cells were harvested.

### 2D gels

*B*. *subtilis* cultures were grown to OD 0.3, then treated with 0.2% NaAzide to arrest growth. 20 mg of cells were then suspended in low-melt agarose plugs (0.5%) as previously described [45]. Lysis was performed in 2 mg/ml lysozyme for 16 hours at 37°C. Protein was removed via incubation with 5 mg/ml proteinase K, 5% sarkosyl, 0.5 M EDTA for 4 hours at 37°C. Proteinase K was then removed by 8 successive 4 hour washes in TE at 4°C. DNA was digested overnight in plugs equilibrated in 1x CutSmart buffer plus a 0.5 μl of each of the indicated enzymes (NEB). DNA was subjected to 2-dimensional electrophoresis and Southern blotting as previously [46]. Probes for Southern blots were generated via random priming of gel-extracted PCR products corresponding to the *lacZ* region or the *rrn16S-23S* rRNA regions. Probes were radioactively labelled using α-^32^P-dATP. Hybridization images were generated on X-ray film or with phosphor screens (GE Healthcare). Y-arcs were quantified by conforming a single lane to the shape of the entire arc in ImageQuant software (GE Healthcare). This yielded a histogram consisting of approximately 450 data points along the arc.

### Strain constructions

Strains used in this publication are listed in Table 1. The ImmR protein, which is coded for by the mobile genetic element ICE*Bs*1, tightly represses P_*xis*_ [47]. We introduced the P_*xis*_-*lacZ* constructs into strains that either harbored (Trx-) or were cured of (Trx+) ICE*Bs*1. To produce the PcrA degron strains, genomic DNA from strain HM448 was transformed into strains harboring the P_*xis*_-*lacZ* constructs prior to selection on MLS for the P_*spank*_ *-sspB* construct. The *pcrA-ssrA* allele from HM448 was then transformed into the resulting strains as a second transformation.

**Table 1:** Strains used in this study.

HM #	Genotype	Reference
HM1	*trpC2 pheA1*; wild type	
HM224	*pcr*::P_spank_ RBS 3x*myc*-*pcrA* (*cat)*	[51]
HM448	pcrA-ssrA*1093-cat thrC::*Pspank-sspB*::*mls*	This Study
HM292	*rpoC-gfp*::*mls*	[52]
HM862	*pcrA*::*Pspank RBS 3xmyc-pcrA (cat) thrC*::*Pxis-lacZ*::*mls (HO*, *OFF)*	This Study
HM863	*pcrA*:: *Pspank RBS 3xmyc-pcrA (cat) thrC*::*Pxis-lacZ HO*::*mls (HO*, *ON)*	This Study
HM864	*pcrA*::*Pspank RBS 3xmyc-pcrA (cat) thrC*::*Pxis-lacZ CD*::*mls (HO*, *OFF)*	This Study
HM865	*pcrA*::*Pspank RBS 3xmyc-pcrA (cat) thrC*::*Pxis-lacZ CD*::*mls (HO*, *ON)*	This Study
HM876	pcrA-ssrA*1093-cat thrC::*Pspank-sspB spec thrC*::*Pxis-lacZ*::*mls (HO*, *OFF)*	This Study
HM877	pcrA-ssrA*1093-cat thrC::*Pspank-sspB spec ICEBs10 thrC*::*Pxis-lacZ*::*mls (HO*, *ON)*	This Study
HM878	pcrA-ssrA*1093-cat thrC::*Pspank-sspB spec thrC*::*Pxis-lacZ*::*mls (CD*, *OFF)*	This Study
HM879	pcrA-ssrA*1093-cat thrC::*Pspank-sspB spec ICEBs10 thrC*::*Pxis-lacZ*::*mls (CD*, *ON)*	This Study
HM315	*amyE*::P_*spank*_ *-sspB dnaC-ssrALGG*	[48]
HM490	pcrA-ssrA*1093-cat thrC::*Pspank-sspB*::*mls recF*::*spec*	This Study
HM1110	*amyE*::P*spank-sspB spec thrC*:: P_*spank*_ *-hisC952* (HO)	This Study
HM1111	*amyE*::P*spank-sspB spec thrC*:: P_*spank*_ *-hisC952* (CD)	This Study
HM1117	pcrA-ssrA*1093-cat amyE::*Pspank-sspB spec thrC*:: *Pspank-hisC952*::*mls (HO)*	This Study
HM1118	pcrA-ssrA*1093-cat amyE::*Pspank-sspB spec thrC*:: *Pspank-hisC952*::*mls (CD)*	This Study
HM1020	pcrA-ssrA*1093-cat thrC::*Pspank-sspB*::*mls Pspank-sspB*::*mls amyE*::*Pspank*::*spec*	This Study
HM1021	pcrA-ssrA*1093-cat thrC::*Pspank-sspB*::*mls Pspank-sspB*::*mls amyE*::*Pspank-myc-pcrA*::*spec*	This Study
HM1115	pcrA-ssrA*1093-cat thrC::*Pspank-sspB*::*mls Pspank-sspB*::*mls amyE*::*Pspank-myc-pcrA*669::*spec*	This Study
HM1116	pcrA-ssrA*1093-cat thrC::*Pspank-sspB*::*mls Pspank-sspB*::*mls amyE*::*Pspank-myc-pcrA K37A Q254A*::*spec*	This Study

### Survival assays

Single colonies were grown in liquid LB and grown to OD 0.5, then serially diluted at a 1:10 ratio prior to plating.

### ChIP-qPCR analysis

Polyclonal rabbit anti-DnaC antibodies were used for ChIP of native DnaC [5,48]. Mouse monoclonal anti-Myc antibody purchased from Invitrogen was used for anti-Myc-PcrA ChIP experiments (Product Number 13–2500). Polyclonal rabbit anti-GFP antibodies were used for RpoC-GFP ChIP experiments. DNA samples for ChIP were prepared essentially as previously described [5,48]: *Bacillus subtilis* cells were grown in LB medium as described. Cells were crosslinked with formaldehyde at a final concentration of 1% v/v. Following 20 minutes of incubation at room-temperature, reactions were quenched with glycine, and cells were pelleted, washed once in 1x PBS, pelleted again, then frozen at -80C. Pellets were re-suspended in 1.5 ml solution A (20% sucrose, 50 mM NaCl, 10 mM EDTA, 10 mM Tris pH 8.0), plus 1 mg/ml lysozyme, and 1 mM AEBSF. Following a 30 minute incubation at 37°C, lysates were sonicated on a Fisher sonic dismembrator (Fisher FB120) for 40 seconds (10 seconds on, 10 seconds off), at 30% amplitude. Lysates were spun at 8k rpm for 15 min at 4°C in microcentrifuge tubes, and the supernatant cell extract was transferred to fresh tubes and frozen at -80C.

ChIP was performed by adding 12 μl anti-Myc or 1 μl anti-DnaC antibody to 1 ml aliquots of extract, then incubating over-night at 4°C in an end-over-end nutator. Antibody-bound protein:DNA complexes were precipitated using protein A sepharose beads (GE 45000143), decrosslinked over-night at 65°C, then purified by phenol:chloroform extraction and ethanol precipitation.

qPCR analysis was performed on a Bio-Rad CFX connect (Product Number 1855201) using Sso Advanced SYBR green master mix (product number 1725262). Primer pairs include HM192 (5`-CCGTCTGACCCGATCTTTTA-3`) and HM193 (5`-GTCATGCTGAATGTCGTGCT-3`) which amplify the low conflict region *yhaX*, HM80 (5`-AGGATAGGGTAAGCGCGGTATT) and HM81 (5`-TTCTCTCGATCACCTTAGGATTC-3`) which amplify the *rrn23S* repeat of rRNA gene repeats, HM766 (5`-GCT GGG AGA GCA TCT GCC TT-3`), HM767 (5`-CCAACCTACTGATTACAAGTCAGTTGCTCTA-3`) which amplify between Threonine and Valine tRNA genes at 4 repeats, HM892 (5`-CATGAAAAAGCTCGGCAAAG-3`) and HM893 (5`-TGGAATCTTACGCAAAAACAAA-3`) which amplify within the *rplGB* gene, HM803 (5`-TGTTTTGCGGAGAGGTTCTT-3`) and HM804 (5`-CGGGCCGTACGTATTAAAAA-3`) which amplify within the *dltA* gene, and HM902 (5`-CGGGGTCAGCTACATTATGG-3`), and HM903 (5`-AGACATATGCCAGCGATTCC-3`) which amplify within the *dltB* gene. HM770 (5`-TCTCCAGCTGTGATAAACGGTA-3`) and HM771 (5`-AAAACGGCATTGATTTGTCA-3`) which amplify within the *dnaK* gene, HM952 (5`-GGTGTAAACGAACGTCAATTCCGCAC-3`) and HM953 (5`-AGCTTGTACACAACGTTATCAAGACGAGAATC-3`) which amplify within the *rpsD* gene, and HM954 (5`-GAAGAAAAAGTGAATGAGCTGCTGAAGGAA-3`) and HM955 (5`-AATGTCTTCGCTCTCAAAAAACTCAATCAAACG-3`) which amplify within the *yutJ* gene. qPCR analysis was conducted for both *yhaX* and test DNA species in both input and ChIP samples. Final fold enrichment was calculated as (Test DNA in ChIP Sample/Test DNA in Input Sample)/(*yhaX* in ChIP sample/*yhaX* in Input sample).

### ChIP-Seq analysis

ChIP DNA samples were analyzed first using qPCR to validate that a given sample is representative. DNA samples were then processed and sequenced by the University of Washington High Throughput Sequencing Genomics Center, on an Illumina Next-Seq.

### FASTQ file analysis

Approximately 750k paired-end Illumina Next-Seq reads per sample were mapped against the genome of *B*. *subtilis* strain JH642 (GenBank: CP007800.1) using Bowtie 2 with the—no-mixed option. This option prevents unpaired alignments, such that only reads that aligned uniquely at both ends were mapped [49]. As discordant mapping was minimal and did not significantly alter the resulting profile, discordant mapping was active. The resulting. sam file was processed by SAMtools, view, sort, and mpileup functions [50], to produce wiggle plots. We tested the effect of removing PCR-based and optical duplicates using Picard v1.3 and found that the same gene regions were identified in subsequent analyses, but at a slightly lower signal:noise ratio. Therefore, in the presented data sets, duplicates were not removed. Myc-PcrA ChIP-Seq data and antibody control data were first normalized to input samples (signal in the ChIP sample minus the signal in the corresponding input sample). Normalized antibody control IP (Mock IP) data representing non-specific signal enrichment was subtracted from the normalized ChIP signal. For DnaC ChIP-Seq, ChIP samples were first normalized to inputs (ChIP minus input). The normalized + PcrA DnaC ChIP sample signal was then subtracted from the normalized—PcrA DnaC ChIP signal at each nucleotide position, establishing the differential signal.

ChIP-Seq data were quantified as follows: for each gene and intergenic region, the maximal signal, average signal, area under the curve, and area under the curve divided by total gene length (normalized area under the curve) were calculated. Local background was independently determined for regions proximal to *oriC* (the 0–300k nucleotide region, where background signal is slightly higher due to higher chromosomal copy number), or distal to *oriC*, by calculating the average maximal signal in ~100 kb regions that were devoid of peaks. Genes containing a maximum signal of more than 5-fold above background were called as peak-containing regions.

## Supporting Information

S1 Fig Normalization of ChIP signal to the *yhaX* locus allows for robust sample comparison and preserves trends in ChIP data identified at the *lacZ* locus. Here data are presented as individual non-normalized sample isolates, averaged non-normalized samples, and average normalized samples (to totals and *yhaX*). Because we normalized using a control locus (*yhaX*) we did not normalize the amount of template DNA added to each reaction, leading to apparent variability between non-*yhaX*-normalized replicates. A) DnaC ChIP in the presence or absence of transcription, B) DnaC ChIP in the presence or absence of PcrA, C) PcrA and DnaC ChIP-qPCR signal-/+ transcription or-/+ PcrA (DnaC only) demonstrates that target protein association with *yhaX* does not change between experimental conditions, making *yhaX* an ideal normalization locus.
(EPS)

S2 Fig RNA polymerase association does not decrease following replication inhibition. RpoB ChIP-qPCR indicates that RNA polymerase association with conflict regions is not ablated by 30 minute HPura treatment (which inhibits replication). However, HPUra treatment does cause a major decrease in DnaC association at the same locus as demonstrated in Fig 1D.
(EPS)

S3 Fig A second Y-arc confirms trends apparent on the EagI/ApaLI digestion of the *lacZ* gene region. Here the 2D gel data displayed in fig 3B, bottom right panels, are shown again, but with the second Y-arc emanating from the undigested spot fully displayed. Here the left edge of the Y-arc is free from background signal. This allows for an improved view of the *lacZ* gene region where replication intermediate signal decreases after PcrA is depleted. White arrows indicate the approximate location of the 3`end of the *lacZ* gene within the second Y-arc.
(EPS)

S4 Fig N-terminally Myc-tagged PcrA rescues cell death caused by depletion of endogenous PcrA. PcrA degron strains harboring an empty vector (left) or a second, N-terminally Myc-tagged copy of PcrA, integrated at *amyE* (right) were plated on media lacking IPTG (top), or including IPTG (bottom). Cells harboring the Myc-PcrA allele were viable following depletion of endogenous PcrA, demonstrating the functionality of the tagged protein.
(EPS)

S5 Fig Replisome stalling increases at chromosomal regions following PcrA depletion. Candidate gene regions from DnaC ChIP-Seq (which are also quantified by qPCR in Fig 6 and S9 Fig) are shown in detail, either before (blue) or after (red) PcrA depletion. Sequencing coverage is indicated on the left, the gene or gene regions are identified at the top of each box, and the location(s) of the gene(s) are indicated below. Gene orientation relative to replication is denoted “HO” for head-on genes, and “CD” for co-directional genes.
(EPS)

S6 Fig DnaC ChIP-Seq prior to and post-normalization. A) Wiggle files for DnaC ChIP prior to (left) and following (right) PcrA depletion, as well as total (input) samples for both conditions, and ChIP minus total normalizations. B) Final normalization of the DnaC ChIP-Seq data set. Here the total normalized DnaC ChIP-Seq signal +PcrA (S5A Fig, bottom left), was subtracted from—PcrA condition signal (S5A Fig, bottom right).
(EPS)

S7 Fig PcrA ChIP-Seq prior to and post-normalization. A) Wiggle files for PcrA ChIP, total (input), and ChIP minus total normalization. B) Wiggle file for Mock IP, total (input), and Mock IP minus total normalization. C) Magnified view of one chromosomal region encoding several rDNA repeats following total normalization. D) Final normalization of the PcrA ChIP-Seq data set on a global level (right), and within the first 200k nucleotides. * Denotes the position of a single tRNA gene.
(EPS)

S8 Fig Replication intermediates accumulate in rRNA genes following PcrA depletion. To test for potentially slow replication through rDNA genes after PcrA depletion, chromosomal DNA was digested using KpnI and EagI restriction enzymes which cut at the same position in all rRNA genes, causing all 10 rRNAs to run together during 2D gel electrophoresis. A) Restriction digest map showing the 16S-23S rRNA fragment that was probed against during 2D gel analysis. B) 2D gels for rRNA gene fragments in the presence or absence of PcrA. The relative amount of DNA loaded, as indicated by quantification of the non-replicating 1N spot, is indicated at the top right. The appearance of an arc of replication intermediates following PcrA depletion indicates replication slowing/stalling in this region.
(EPS)

S9 Fig RecF is not required for the increase in replisome stalling observed after PcrA depletion. Replisome stalling at *rrn23S* was measured by DnaC ChIP-qPCR in the presence (left) or absence of *recF* (right), and before (black) or after PcrA depletion (white). The equivalent increase in DnaC association following PcrA depletion in the presence or absence of RecF suggests that the viability of the PcrA degron Δ*recF* strain, following PcrA depletion, is not due to a reduction in replication fork stalling.
(EPS)

S10 Fig Additional peaks identified via DnaC ChIP-Seq also show increased replisome stalling after PcrA depletion when assayed using ChIP-qPCR. Replisome stalling, as measured by DnaC ChIP-qPCR, was measured for three additional gene regions (all head-on) identified in Fig 5. These locations each show increased DnaC association after PcrA depletion, verifying that PcrA mitigates conflicts in these regions.
(EPS)

S11 Fig Normalization of ChIP signal to the *yhaX* locus allows for more accurate sample comparison. ChIP-qPCR data showing A) DnaC association with experimental locus *rrn23S* as individual non-normalized sample isolates, B) DnaC association with control locus *yhaX* as individual samples, or C) averaged samples normalized only to input (total samples) and average normalized samples (to totals and *yhaX*). Trends in the data are observable prior to, as well as after normalization. However, normalization controls for sample loading, and ChIP efficiency, thereby contributing to a more accurate and consistent measurement of protein association with test locus *rrn23S*.
(EPS)

S12 Fig RNA polymerase beta and beta`subunits shows similar association patterns with genomic loci. To measure relative RNA polymerase occupancy, ChIP-qPCR of RNA polymerase subunits beta (RpoB), or beta`(RpoC-GFP), were analyzed for their association with four genomic loci. RpoC-GFP was immunoprecipitated using anti-GFP antibodies, whereas RpoB was immunoprecipitated with a native RpoB monoclonal antibody (Abcam ab12087). The *rrn23S*, and *rplGB* loci are co-directional, and the *dltA*, and *rpsD* loci are head-on to replication.
(EPS)

S13 Fig PcrA essentiality is linked to its activity in conflict mitigation. A) Cells exhibit equal plating efficiency in the presence of PcrA (left panel), regardless of whether they harbor the non-transcribed co-directional *hisC* allele (lanes 3 and 4, left panel) or the non-transcribed *hisC* head-on allele (lanes 3 and 4, left panel). Also, the non-induced PcrA degron system does not affect viability relative to strains lacking the PcrA degron (lanes 2 and 4, versus 1 and 3, respectively). In these strains, IPTG addition (right panel) leads to the simultaneous induction of *hisC*, and induction of the *sspB* adaptor protein gene. Hence IPTG addition causes cell death in strains harboring the PcrA degron (lanes 2 and 4, both panels), but not in strains that lack the complete degron system (lanes 1 and 3, both panels). (Control strains in lanes 1 and 3 possess the sspB adaptor protein, but lack the *pcrA-ssrA* allele). The strain harboring the PcrA degron system and the transcribed co-directional *hisC* allele shows less sensitivity to partial depletion of PcrA, than the equivalent strain with a transcribed head-on *hisC* allele (right panel, lanes 2 vs. 4). B). Quantification of cell survival after partial PcrA depletion (left) or near-complete PcrA depletion (right). N ≥ 3 for all conditions. * indicates that no colonies were detected.
(EPS)

S1 Table Peaks in the DnaC ChIP-Seq data set. Here the quantitative ChIP-Seq data for each chromosomal region where DnaC enrichment increases to 5-fold above background following PcrA depletion are reported. Data include the maximal and average number of reads within each defined gene or intergenic region. We also report the area under the curve, and area under the curve divided by the total regions length for each region.
(TXT)

S2 Table DnaC ChIP-Seq quantification for all chromosomal regions. (TXT)

S3 Table Peaks in the PcrA ChIP-Seq data set. Here the quantitative ChIP-Seq data for each chromosomal region where DnaC enrichment increases to 5-fold above background are reported. Data include the maximal and average number of reads within each defined gene or intergenic region. We also report the area under the curve, and area under the curve divided by the total regions length for each region.
(TXT)

S4 Table PcrA ChIP-Seq quantification for all chromosomal regions. (TXT)
